# UPLC Q-TOF/MS-Based Metabolic Profiling of Urine Reveals the Novel Antipyretic Mechanisms of Qingkailing Injection in a Rat Model of Yeast-Induced Pyrexia

**DOI:** 10.1155/2013/864747

**Published:** 2013-06-06

**Authors:** Xiaoyan Gao, Mingxing Guo, Long Peng, Baosheng Zhao, Jiankun Su, Haiyu Liu, Li Zhang, Xu Bai, Yanjiang Qiao

**Affiliations:** ^1^Science Experiment Center for Traditional Chinese Medicine, Beijing University of Chinese Medicine, No. 11 North Third Ring Road, Chaoyang District, Beijing 100029, China; ^2^Waters Technologies (Shanghai) Ltd., No. 1378 Zhangdong Road, Shanghai 201203, China; ^3^Key Laboratory of TCM-Information Engineering of State Administration of TCM, Beijing Key Laboratory for Basic and Development Research on Chinese Medicine, Beijing University of Chinese Medicine, No. 6 Zhonghuan South Road, Wangjing, Chaoyang District, Beijing 100102, China

## Abstract

Fever is one of the most common clinical symptoms of many diseases. Qingkailing (QKL) injection is widely used in China as a clinical emergency medicine due to its good antipyretic effects. It is a herbal formula which is composed by eight kinds of traditional Chinese medicines (TCM). As a kind of typical multiple constituents and multiple actions of TCM, it is very difficult to elaborate the antipyretic mechanism by conventional pharmacological method. Metabonomics technique provides beneficial tool for this challenge. In this study, an ultra performance liquid chromatography quadrupole time-of-flight mass spectrometry (UPLC Q-TOF/MS) metabonomics method was developed to explore the changing process of biochemical substances in rats of yeast-induced pyrexia. Partial least squares discriminate analysis (PLS-DA) was used to distinguish the normal control group, the pyrexia model group, and the pyrexia model group treated by QKL injection. The potential biomarkers related to pyrexia were confirmed and identified. MetPA was used to find the possible metabolic pathways. The results indicated that the antipyretic effect of QKL injection on yeast-induced pyrexia rats was performed by repairing the perturbed metabolism of amino acids.

## 1. Introduction

Fever is one of the most common clinical symptoms of many diseases, such as infection, inflammation, trauma, and metabolic disorders. Fever was defined as a state of elevated core temperature, which is often, but not necessarily, part of the defensive responses of multicellular organisms (host) to the invasion of live (microorganisms) or inanimate matter recognized as pathogenic or alien by the host [1]. Although some kinds of fever were beneficial to the host, it is not without its ill-effects, especially in cases of very high fevers, or when high fever is associated with comorbidities such as severe sepsis and preexisting cardiopulmonary disease [2]. So, antipyretic is still necessary in these situations. The ordinary antipyretic medications, the nonsteroidal anti-inflammatory drugs (NSAIDs), for example, ibuprofen, aspirin, and acetaminophen, are used primarily as pain relievers or anti-inflammatory. More and more debates over the appropriate use of such medications with the serious side effects of NSAIDs have been increasingly reported [3–8].

Traditionally, plants/herbs with antipyretic properties are commonly used in China. More than one thousand years ago, fever was discussed in *Shanghanlun*, a classic book about the basic theory of TCM, written by Zhongjing Zhang, a medical sage respected by the Chinese. Since then, Chinese medicines are still in use as antipyretic. In the large number of Chinese medicine preparations with antipyretic action, QKL injection is focused on for its significant effect of antipyretic action and larger market share. QKL injection is prepared from eight medicinal materials or their extracts, including *Radix Isatidis*, *Flos Lonicerae*, Fructus Gardeniae, Cornu Bubali, *Concha Margaritifera*, Baicalin, Cholic acid and Hyodeoxycholic acid [9]. It is commonly used in clinical practice for the treatment of high fever, upper respiratory inflammation, viral encephalitis, hepatitis, stoke, cerebral thrombosis, tonsillitis, tracheitis [10, 11]. Though QKL injection has been widely used to treat the fever patients in clinic in China, few literatures have reported about the detailed antipyretic mechanism. For one reason, TCM and preparation complexity and variability present challenges for researchers. For another reason, the conventional studies on TCM pharmacological mechanism have faced many difficulties.

Metabonomics is a new platform of systems biology, defined as the quantitative measurement of the dynamic multiparametric metabolic responses of living systems to pathophysiological stimuli or genetic modifications [12]. Metabonomics makes an impact at several points in the drug mechanism research: multitarget validation, multiple mechanism integration, and comprehensive evaluation [13–15]. The application of metabonomics has dramatically increased in the fields of explanation of medicines mechanism and evaluation of drug efficacy and toxicity [16–18]. Published results indicated that metabonomics presented the potential capability to improve our understanding of TCM like QKL injection [19–22]. Recently, some advanced instruments and bio-information analysis techniques enable us to perform the simultaneous analysis of a large number of metabolites [23–26]. UPLC-Q-TOF/MS system can provide accurate mass of fragments, precursor ions, and neutral loss information, and it has a larger peak capacity and better resolution and sensitivity. Metabonomics technique combined with advanced instruments and appropriate data processing method provides support to us for explaining the antipyretic mechanism of QKL injection.

In the present study, yeast-induced fever is adopted as pathogenic fever model [27]. This type of fever leads to an intense inflammatory reaction caused by ulceration at the injection site. This model is suitable for studying the effects of some antipyretic medicines such as QKL injection [28–31]. In this paper, a metabonomics method was employed to explore the mechanism of the antipyretic effects of QKL injection.

## 2. Materials and Methods

### 2.1. Chemicals and Reagents

QKL injection was purchased from Yabao Beizhongda (Beijing) Pharmaceutical Co., Ltd. (no. 012907A, China). Yeast was obtained from Mauri Food Co., Ltd. (Heibei, China). LC-grade methanol and acetonitrile were acquired from Baker Company (Baker Inc., USA). Ultra high-purity water was prepared by Millipore-Q SAS 67120MOLSHEIM (France). Formic acid (no. 7000027413) was obtained from Sigma Chemical Co., Ltd. (St. Louis, MO, USA).

### 2.2. Animals and Sample Collection

The protocol of the study was approved by the Ethics Committee of Beijing University of Chinese Medicine. All procedures and care of the rats were in accordance with the institutional guidelines for animal use in research. Male Sprague-Dawley rats (200 ± 20 g) were obtained from Beijing Weitonglihua Laboratory Animal Technology Co., Ltd. (Beijing, China). Before use in the study, all the rats were acclimated for five days in a controlled room with temperature (23 ± 2°C), humidity (60 ± 5%), and a light/dark cycle of 12 h. Then they were transferred to individual metabolism cages and allowed to acclimatize for an additional three days. During that time, the rats' rectal temperatures were measured three times per day using a digital thermometer for the regular rhythm of body temperatures. The rats with a temperature difference that was greater than 0.5°C were excluded. Twenty-four qualified rats were selected and divided into three groups randomly: the normal control group (CG), the pyrexia model group (PG), and the pyrexia model group treated by QKL injection (TG). After the start of the experiment, the rats of PG and TG were subcutaneously injected with a 20% aqueous suspension of yeast (15 mL/kg) in the back of rats. The rats of CG were similarly given an equal volume of 0.9% saline. One hour later, the rats of TG were injected with 4.2 mL/kg QKL injection in the tail vein. The rats of CG and PG were similarly injected with an equal volume of 0.9% saline in the tail vein. Urine was collected before yeast was injected and at different time-points, including 4 h, 8 h, 12 h, 24 h, 36 h, 48 h, 60 h, and 72 h after QKL was injected. The rectal temperature was also measured during that time. Urine was centrifuged at 14000 rpm for 10 min at 4°C, and the supernatant was collected and stored at −20°C.

### 2.3. Urine Sample Preparation

The urine samples were thawed at room temperature. The supernatant was diluted at a ratio of 2 : 1 with distilled water, vortex mixed and filtered through a 0.22 *μ*m filter membrane. A 2 *μ*L sample was injected into the UPLC Q-TOF/MS system for analysis.

### 2.4. UPLC-Q-TOF/MS Analysis

Waters Acquity Ultra Performance LC system (Waters Corp., Milford, MA, USA) was performed to separate the metabolites, which was equipped with an HSS T3 column (2.1 mm × 100 mm, 1.7 *μ*m, UK). The column temperature was set to 45°C and the gradient elution program started with 99% solvent A and 1% solvent B (solvent A: 0.1% formic acid in water; solvent B: acetonitrile modified by the addition of 0.1% formic acid). The column was eluted with a linear gradient of 99%–60% A over 0.5 to 10 min, 60%–1% A over 10 to 11 min, 1% A was held for 1 min and then returned to 99% A over 12.0 to 15.0 min at a flow rate of 0.45 mL/min.

A Xevo G2-Q-TOF (Waters MS Technologies, Manchester, UK) was performed on mass spectrometry with an electrospray ionisation source. The positive ion mode was performed. Data were collected from m/z 50 to m/z 1200. The capillary and cone voltage were set at 3.0 kV and 45 V, respectively. The desolvation gas was set at 800 L/h at a temperature of 450°C, the cone gas was set at 30 L/h, and the source temperature was set at 120°C. The Leucine enkephalin was used as the lock mass solution in accurate mass measurement.

### 2.5. Data Analysis

The statistical analysis of the rectal temperature was performed by SPSS 17.0 (SPSS Inc., Chicago, IL, USA).

Mass spectrometry data was processed using Waters Markerlynx XS software. The UPLC-MS data were detected and noise-reduced in both the UPLC and MS domains such that only true analytical peaks were further processed by the software. A list of intensities (chromatographic peak areas) of the peaks detected was then generated for the first chromatogram, using the *t*_*R*_-*m*/*z* data pairs as identifiers. The data was analyzed further with PLS-DA. For the identification of potential biomarkers, the following databases had been used: HMDB (http://www.hmdb.ca/), KEGG (http://www.genome.jp/kegg/), Massbank (http://www.massbank.jp/), Chemspider (http://www.chemspider.com/), and METLIN (http://metlin.scripps.edu/). Ingenuity pathway analysis (IPA) was performed with MetPA (http://metpa.metabolomics.ca./MetPA/faces/Home.jsp) based on database sources including the KEGG, HMDB, SMPD (http://www.smpdb.ca/), and METLIN to identify the affected metabolic pathways analysis and visualization.

## 3. Results and Discussion

### 3.1. Efficacy of QKL Injection as Antipyretic

**Figure 1:**
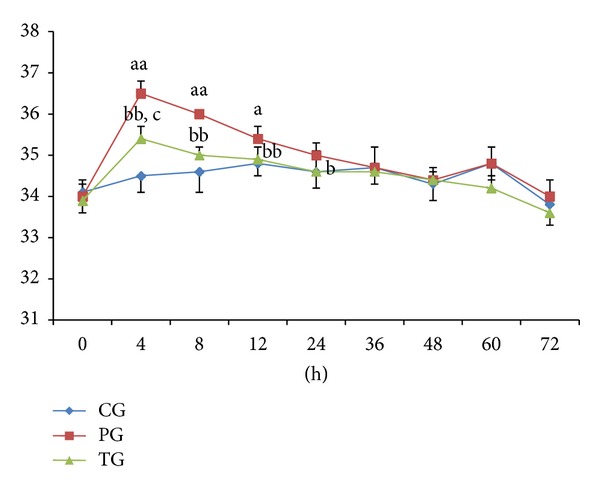
Results of rectal temperature in CG, PG, and TG. Data were expressed by independent sample *t*-test. _ _^aa^ *P* < 0.01, _ _^a^ *P* < 0.05 compared with CG. _ _^bb^ *P* < 0.01, _ _^b^ *P* < 0.05 compared with PG. _ _^c^ *P* < 0.05 compared with CG.

To monitor the temperature changes, the rectal temperatures of the rats in each group before and after QKL injection administrated were tested. As showed in Figure 1, at the time-point of 4 h, the body temperature was elevated by 2.0 ± 0.5°C (*P* < 0.01, versus CG) in PG, indicating that the yeast-induced pyrexia model was succeed. At the same time-point, for TG, the body temperature dropped by 1.1 ± 0.4°C (*P* < 0.01, versus PG) after QKL injecting, demonstrating that QKL injection had significant antipyretic effects on yeast-induced fever rats. At the time-point of 8 h after QKL was injected, the body temperature reached the normal level (*P* > 0.05, versus CG).

### 3.2. Multivariate Data Analysis

UPLC Q-TOF/MS was used to collect the urine information. The detailed information of the UPLC-Q-TOF/MS analysis and validation was shown in Supplementary Materials (Text S1 and Figure S1) available online at http://dx.doi.org/10.1155/2013/864747.

**Figure 2:**
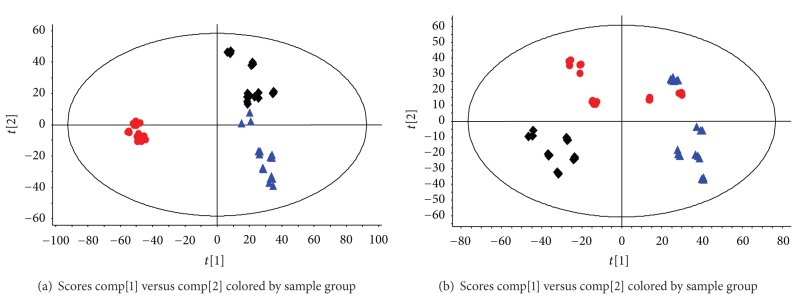
PLS-DA score plot obtained from CG (▲), PG (◆), and TG (●) at 4 h (a) and 8 h (b).

A method of PCA was used for unsupervised multivariate data analysis. It is well known that drug-induced ingredients will affect the results of the cluster analysis, even deceptively. In the previous study of our laboratory, more than 40 organic ingredients were found in QKL injection, among which, bile acid, hyodeoxycholic acid, baicalin, and geniposide were higher-content compounds [32, 33]. In this paper, the high contents of drug-induced components and their metabolites were deducted before using the multivariate data analysis (see Supplementary Materials, Table S1). All of these ingredients were also verified by Thermo Accela HPLC-LTQ/Orbitrap coupled LC-MS system (Thermo Fisher Scientific, Bremen, Germany) (see Supplementary Materials, Figure S2).  Figure 2 showed the PLS-DA score plot among CG, PG, and TG at the time-points of 4 h and 8 h. The result demonstrated that the metabolites in the pyrexia model rats were disturbed by the fever pathological state (PG versus CG) and the treatment rats which were gradually returned to normal based on the plots of TG were gradually close to the plots of CG (8 h versus 4 h). Interestingly, the results were almost in accordance with the variation of body temperature. We could speculate that the changes of body temperature were highly related to the disturbance of the endogenous components. Accordingly, the time-point of 4 h after QKL injection was administered was selected to reveal the antipyretic mechanism of QKL injection due to the plots separated clearly among the three groups.

### 3.3. Detection and Elucidation of Biomarker Candidates

To reveal the antipyretic mechanism of QKL injection, a PLS-DA loading plot was used to find potential biomarkers among CG, PG, and TG (see Supplementary Materials, Figure S3). The potential biomarkers were chosen based on two rules: for one rule, the VIP values (variable importance in the projection) from the PLS-DA model must be higher than 2; for the other rule, the compound changing trends of TG and PG must be opposite. Twenty-one metabolites were selected as potential biomarkers to characterize the antipyretic mechanism of QKL injection (Table 1). The structural elucidation of biomarker candidates is a challenging task. The detailed identification procedure of the biomarkers was shown in Supplementary Materials (Text S2 and Figure S4).

### 3.4. Explore the Antipyretic Mechanism of QKL Injection

**Figure 3:**
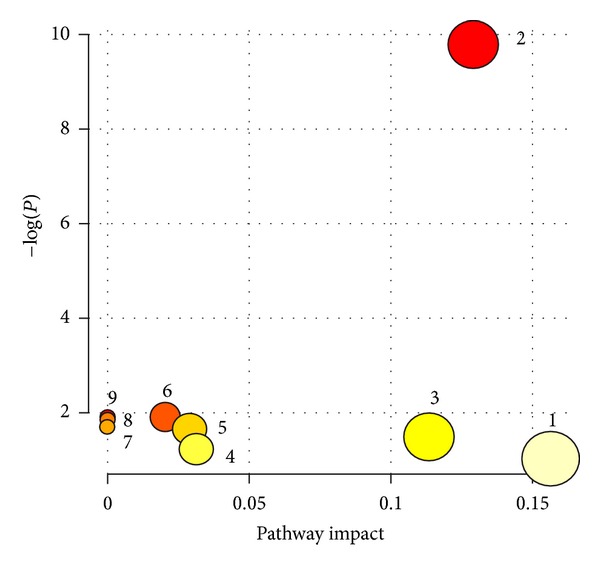
Summary of pathway analysis. (1) Tryptophan metabolism; (2) arginine and proline metabolism; (3) alanine, aspartate and glutamate metabolism; (4) glycine, serine, and threonine metabolism; (5) butanoate metabolism; (6) pantothenate and CoA biosynthesis; (7) histidine metabolism; (8) aminoacyl-tRNA biosynthesis; (9) beta-alanine metabolism.

**Figure 4:**
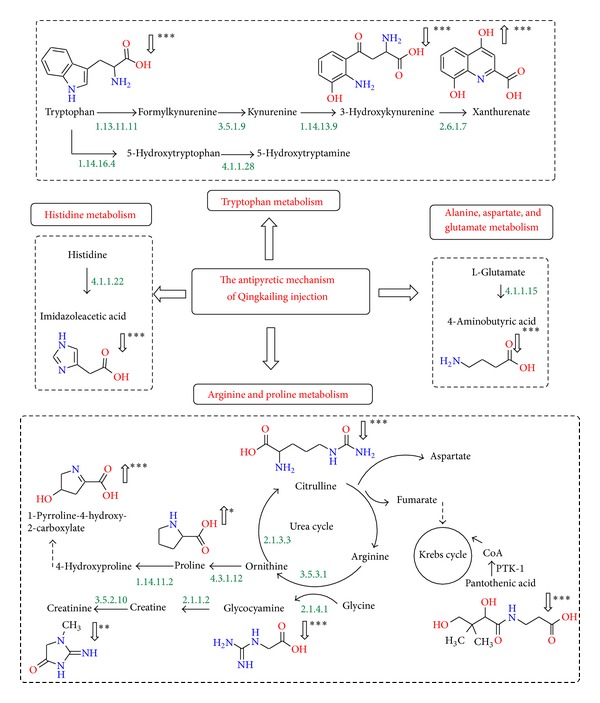
The perturbed metabolic pathways in response to the antipyretic mechanism of QKL injection.

The twenty-one biomarkers were listed in Table 1. As we expected, the changing trends of TG and PG were opposite. After QKL injection was administrated, these differences of metabolites may denote their potential as targeted biomarkers for the antipyretic mechanism of QKL injection. More detailed analysis of pathways and networks influenced by QKL injection was performed by ingenuity network analysis (IPA). In order to identify possible pathways affected by QKL injection, biomarkers contributing to the separation of the CG, PG, and TG were analyzed using MetPA. MetPA is a free, web-based tool that combines result from powerful pathway enrichment analysis with the pathway topology analysis to help researchers identify the most relevant pathways involved in the conditions under study [34–36]. It is well known that repairing in more important positions of a network will arouse a more active impact on the pathway than repairing occurring in marginal or relatively isolated positions. Metabolic pathway analysis with MetPA revealed that these potential biomarkers, which were important for the antipyretic mechanism of QKL injection, were responsible for tryptophan metabolism; arginine and proline metabolism; alanine, aspartate, and glutamate metabolism, and so on. MetPA generated 9 networks (Figure 3, Table S2 of Supplementary Materials), demonstrating that the repaired amino acids metabolism was the antipyretic mechanism of QKL injection (Figure 4).

#### 3.4.1. Tryptophan Metabolism

3-Hydroxykynurenine and xanthurenate were the metabolites of tryptophan. These three biomarkers were closely related to tryptophan metabolism. L-tryptophan is an essential amino acid in human body; it participates in the regulation of protein synthesis and metabolic networks. There are two main ways for the degradation of tryptophan, namely, the kynurenine pathway and 5-hydroxytryptamine (5-HT) pathway [37, 38]. Some studies have shown that the monoamine neurotransmitters in the centre participate in the regulation of body temperature [39]. When 5-HT was injected into the intraventricular, it could cause fever [40, 41]. The elevated tryptophan in PG might indirectly reflect the increasing of 5-HT in the central, which leads to fever. Meanwhile, 3-hydroxykynurenine was also increased in the pathway, but xanthurenate, the metabolite of 3-hydroxykynurenine, dropped in PG. The decrease might be caused by the change of enzyme activity between 3-hydroxykynurenine and xanthurenate, which led to generate a large number of 5-HT. After QKL injection was administrated, the content of tryptophan decreased significantly, indicating that by enhancing the activity of the enzyme between 3-hydroxykynurenine and xanthurenate, it regulated the hypothalamic thermoregulatory center.

#### 3.4.2. Arginine and Proline Metabolism

Glycocyamine, citrulline, proline, 1-pyrroline-4-hydroxy-2-carboxylate, and 4-aminobutyric acid participated in arginine and proline metabolism. L-arginine is an amino acid that has numerous functions in body. It helps disposal of ammonia, which is used to generate compounds such as nitric oxide, creatine, L-glutamate, and L-proline, and it can be converted to glucose and glycogen if needed [42–44]. The pathway contains urea cycle and creatinine metabolism. Citrulline and glycocyamine were the intermediate and metabolite in urea cycle. In the urea cycle, it generates fumarate which associates with the Krebs cycle. The Krebs cycle is the final pathway of the three major nutrients and it is the hub of carbohydrates, lipids, and amino acid metabolites. As we all know, fever causes the three major nutrients' metabolism disorder. The disorder may be related to the perturbed of arginine metabolic pathway. Meanwhile, the elevated pantothenic acid which is the precursor substance of CoA also proved the enhancement of Krebs cycle. 

Our study found that glycocyamine and citrulline in fevered rats were significantly increased. After QKL injection was administrated, these two substances significantly decreased, indicating that QKL injection had an active impact on the urea cycle. QKL injection might reduce the speed of the urea cycle thus further corrected the metabolic rate of the three major nutrients' metabolism. Studies have shown that arginine plays an important regulatory role in wound healing [45]. Yeast-induced fever is a severe inflammatory response. The increased arginine in administered rats would contribute to the healing of the wound.

Much or all of the pyrrole-2-carboxylate in human urine may be formed from labile precursors; the probable source of pyrrole-2-carboxylate is free hydroxy-L-proline [46]. Proline hydroxylation generates hydroxyproline. Hydroxyproline is the main component of collagen in body [47–49]. When the body's bones or skin is damaged, take yeast stimulation, for example, the collagen metabolism is affected. The decreased pyrrole-2-carboxylate and proline in fever rats demonstrated that collagen metabolism is inhibited. After QKL injection was administrated, the two substances were increased, meaning that QKL injection corrected collagen metabolism. The amount of the serum creatinine is an important indicator in measuring kidney function [50]. QKL injection also plays an important role in the treatment of acute renal failure and nephritis. We found that QKL injection could significantly reduce the amount of creatinine in fevered rats, which also confirmed the role of QKL injection in the treatment of nephritis.

#### 3.4.3. Alanine, Aspartate, and Glutamate Metabolism

4-Aminobutyric acid is an inhibitory neurotransmitter found in the nervous systems and has analgesic effect [51]. The mechanism was for mutual adjustment by GABA_A_ receptor and GABA_B_ receptor. Study found that giving GABA_B_ receptor agonist in the myelin could produce significant analgesic effect. In the fevered rats, the body's stress effect was also reflected by the increasing of 4-aminobutyric acid. After QKL injection was administrated, the 4-aminobutyric acid had a clear downward trend compared with the PG, indicating that QKL injection played an important role in antipyretic and analgesic effects.

#### 3.4.4. Histidine Metabolism

Imidazoleacetic acid is a metabolite of histamine metabolism. Histamine is one of the autacoids and is generated by histidine decarboxylase. When the body is stimulated by physical and chemical factors, the mast cells degranulate and release histamine. Histamine binds with histamine receptor and causes inflammation. Yeast-induced fever is a severe inflammatory reaction. The elevated imidazoleacetic acid in the urine of fevered rats indicated that histamine increased in body, which also proved the pathogenesis of yeast-induced fever. After QKL injection was administrated, imidazoleacetic acid significantly decreased, implying that the body immunity improved. It showed that QKL injection significantly affected the fever caused by inflammation.

## 4. Conclusion

In this paper, a metabonomics approach using UPLC Q-TOF/MS was successfully established to explore the antipyretic mechanism of QKL injection based on the variation of endogenous metabolites. Twenty-one representative biomarkers were found by PLS-DA. By metabolic pathway analysis with MetPA, the repaired amino acids metabolism was identified as the antipyretic mechanism of QKL injection. The antipyretic effect of QKL injection was performed by correcting perturbed metabolism of some metabolites such as tryptophan; arginine and proline; alanine, aspartate, and glutamate; histidine.

## Supplementary Material

The detailed information of UPLC-Q-TOF/MS analysis and validation, identification procedure of the biomarkers, drug-induced compositions and their metabolites, result from pathway analysis with MetPA, typical base peak intensity (BPI) chromatograms, typical MS^1^ to MS^3^ spectra of the metabolite with *m/z* 623.12573, PLS-DA loading plot, and identification of the biomarker tryptophan are presented here.

## Figures and Tables

**Table 1:** Metabolites selected as biomarkers characterized in urine profiles and their change trends.

No.	*t*_*R*_ (min)	*m*/*z*	Metabolites	Trend in PG^a^	Trend in TG^b^	VIP
1	0.5222	104.1074	4-Aminobutyric acid	↑***	↓***	4.73
2	0.5549	118.0867	Glycocyamine	↑***	↓**	5.08
3	0.5648	114.0667	Creatinine	↑	↓**	4.66
4	0.6110	143.0819	Ectoine	↑*	↓*	5.14
5	0.6440	176.0978	Citrulline	↑***	↓***	3.51
6	0.6686	325.1131	Difructose anhydride I	↑**	↓	3.95
7	0.6730	127.0478	Imidazoleacetic acid	↑***	↓***	3.54
8	0.6877	259.0931	3-Methyluridine	↑***	↓***	4.17
9	0.7837	130.0502	1-Pyrroline-4-hydroxy-2-carboxylate	↓	↑***	3.98
10	1.2175	116.0709	Proline	↓**	↑*	3.38
11	1.5638	126.0666	5-Methylcytosine	↑*	↓***	3.07
12	2.6807	227.1029	3-Hydroxykynurenine	↑	↓***	2.18
13	2.7929	220.1183	Pantothenic acid	↑	↓*	5.10
14	2.8259	384.1149	Succinyladenosine	↑***	↓***	6.10
15	3.3189	205.0966	Tryptophan	↑***	↓***	4.84
16	3.4148	206.0452	Xanthurenate	↓***	↑***	6.88
17	3.6484	160.0971	Methylbutyrylglycine	↓***	↑	3.10
18	4.2819	180.0658	Hippurate	↓*	↑	3.53
19	4.8699	162.0553	Indole-3-carboxylic acid	↑	↓**	2.38
20	10.8441	274.2741	Hexadecasphinganine	↑*	↓**	4.06
21	11.3671	812.4414	PE(22:6/20:4)	↑***	↓***	5.39

^a^Change trend compared with CG.

^b^Change trend compared with PG.

The levels of potential biomarkers were labeled with (↓) downregulated and (↑) upregulated (*P < 0.05; **P < 0.01; ***P < 0.001; one-way ANOVA followed by independent samples *t*-test).
