# Supplementary material for: UPLC Q-TOF/MS-Based Metabolic Profiling of Urine Reveals the Novel Antipyretic Mechanisms of Qingkailing Injection in a Rat Model of Yeast-Induced Pyrexia
Source: Evid Based Complement Alternat Med. 2013 Jun 6;2013:864747. doi: 10.1155/2013/864747 (PMC3690234; doi:10.1155/2013/864747)
Supplement: Supplementary file 1 — The detailed information of UPLC-Q-TOF/MS analysis and validation, identification procedure of the biomarkers, drug-induced compositions and their metabolites, result from pathway analysis with MetPA, typical base peak intensity (BPI) chromatograms, typical MS1 to MS3 spectra of the metabolite with m/z 623.12573, PLS-DA loading plot, and identification of the biomarker tryptophan are presented here. [file 864747.f1.doc]

**Supplementary materials**

**UPLC Q-TOF/MS based metabolic profiling of urine reveals the novel** **antipyretic mechanisms of Qingkailing injection in a rat model of yeast-induced pyrexia**

Xiaoyan Gao^a1^, Mingxing Guo^a1^, Long Peng^a^, Baosheng Zhao^a^, Jiankun Su^a^, Haiyu Liu^a^, Li Zhang^a^, Xu Bai^b^, Yanjiang Qiao^c*^

a. Science Experiment Center for Traditional Chinese Medicine, Beijing University of Chinese Medicine, No. 11 North Third Ring Road, Chaoyang District, Beijing 100029, P. R. China

b. Waters Technologies (Shanghai) Ltd., No. 1378 Zhangdong Road, Shanghai 201203, P. R. China

c. Key Laboratory of TCM-information Engineering of State Administration of TCM, Beijing Key Laboratory for Basic and Development Research on Chinese Medicine, Beijing University of Chinese Medicine, No. 6 Zhonghuan South Road, Wangjing, Chaoyang District, Beijing, 100102, P. R. China

^*^Corresponding author: Yangjiang Qiao, Tel: +86 010 84738661; Fax: +86 010 84738661; E-mail: yanjiangqiao@sina.cn

^1^equal contribution

**Text S1. UPLC-Q-TOF/MS Analysis and Validation**

UPLC Q-TOF/MS was used to analysis the urine samples. Figure S1 represented the typical base peak intensity (BPI) chromatograms of the urine samples from CG, PG, and TG at the time-point of 4 h. The differences of metabolic profiles among the three groups indicated that yeast-induced fever in rats caused the changes of endogenous substances, and QKL injection corrected the metabolic profiles of the pyrexia rats.

The applied method was validated by the precision, stability and repeatability of LC-MS system, the post-preparative stability, and the stability of freeze-thaw process. Aliquots (100 μL) of urine samples at the time-point of 0 h were mixed to generate pooled quality control (QC) samples. Eight ions with the retention time and *m*/*z* pairs of 0.58-132.08, 3.80-190.05, 4.29-180.07, 4.87-194.08, 5.47-233.08, 6.61-203.11, 7.79-255.06, 10.84-274.27 were selected. The precision was determined from replicated analysis of the same QC sample over six times. The repeatability was evaluated by analyzing six different QC samples through the same preparation procedure. The post-preparative stability was assessed by analyzing six QC samples stayed at the autosampler (maintained at 4 °C) for 24 h. The stability of freeze-thaw process was evaluated by the QC samples undergone from 1 to 3 freeze-thaw cycles. The system stability was carried out by injecting a QC sample every 10 samples during the whole sample analysis.

The relative standard deviations (RSDs) of the retention time for the precision, repeatability, and system stability were found to be 0-0.42%, 0-0.53%, and 0-0.48%, respectively, while the RSDs for the peak area were within the ranges of 2.38-4.79%, 1.96-4.34%, and 3.09-14.42%, respectively. The relative errors (REs) of the peak area for the post-preparative stability and freeze-thaw cycles were found to be –8.73-9.54% and –14.63-12.78%. These results indicated that the developed method showed good precision, repeatability, and stability for the urine analysis.

Figure S1. Typical base peak intensity (BPI) chromatograms obtained from rat urine in CG (A), PG (B) and TG (C). The differences among the three groups were marked with arrows and columns.

**Text S2. Detailed identification procedure of the biomarkers**

The structural elucidation of biomarker candidates is a challenging task. Here, we take *m/z* 205.0970 as an example to show the procedure of the biomarker identification. Firstly, the accurate mass of the ion was obtained from UPLC Q-TOF/MS. The Formula Predictor was applied to predict the formula of the ion. It gave three candidates with high scores, which were C_9_H_11_N_5_O, C_11_H_13_N_2_O_2_ and C_7_H_16_N_3_O_2_P. Secondly, the online database for example HMDB, METLIN, Massbank, Chemspider and KEGG were searched. In the HMDB, Massbank and KEGG, a metabolite named tryptophan was found. In KEGG and Massbank we also found 3-ethyl-5-phenyl-2,4-imidazolidinedione, 5-ethyl-5-phenyl-2,4-imidazolidinedione, idazoxan, (3S)-1,2,3,9-tetrahydropyrrolo[2,1-b]quinazoline-3,7-diol, their formula was also C_11_H_13_N_2_O_2_. Thirdly, the fragment ions of *m/z* 205.0970 were extracted by MS^E^ technique. Four fragments were found, including *m/z* 188.0706, 146.0605, 118.0653 and 91.0544. These fragments were inferred as the fragment ions of *m/z* 205.0966. Moreover, the ion of *m*/*z* 205 lost 17 to generate a fragment ion of *m*/*z* 188, indicating that the metabolite contains a group of –NH_2_. The structures of the metabolites with *m/z* 205.0970 found in the database were compared. It was found that only tryptophan contains the group of –NH_2_. So, this fragment was preliminarily determined as tryptophan. Meanwhile, the generation of the fragments *m*/*z* 146, 118 and 91 (Figure S4) and the MS information by the HMDB database were also supported our inference. Therefore, the metabolite was tentatively identified as tryptophan. Following the same process, other metabolites were identified.

Figure S4. Fragment pattern of the biomarker tryptophan.

Table S1. Drug-induced compositions and their metabolites in urine of QKL injected rats.

| No. | [M+H]^+^ | t_R_  (min) | Formula | Chemical class | Proposed structure |
| --- | --- | --- | --- | --- | --- |
| 1 | 447.0921 | 7.5020 | C_21_H_19_O_11_ | Baicalin |  |
| 2 | 447.0924 | 5.8439 | C_21_H_19_O_11_ | Baicalin metabolite |  |
| 3 | 623.1245 | 6.5968 | C_27_H_27_O_17_ | Baicalin metabolite |  |
| 4 | 609.1449 | 6.5968 | C_27_H_29_O_16_ | Baicalin metabolite |  |
| 5 | 461.1077 | 8.5796 | C_22_H_21_O_11_ | Baicalin metabolite |  |
| 6 | 433.1121 | 8.5889 | C_21_H_21_O_10_ | Baicalin metabolite |  |
| 7 | 271.0603 | 7.5051 | C_15_H_11_O_5_ | Baicalin metabolite |  |
| 8 | 285.0759 | 11.1044 | C_16_H_13_O_5_ | Baicalin metabolite |  |
| 9 | 519.1133 | 7.1741 | C_24_H_23_O_13_ | Baicalin metabolite |  |
| 10 | 389.1445 | 4.6911 | C_17_H_25_O_10_ | Geniposide |  |
| 11 | 359.1383 | 8.4377 | C_16_H_23_O_9_ | Geniposide metabolite |  |
| 12 | 227.0916 | 4.6927 | C_11_H_15_O_5_ | Genipin |  |
| 13 | 209.0813 | 4.6922 | C_11_H_13_O_4_ | Genipin fragment |  |
| 14 | 551.1981 | 4.6314 | C_23_H_35_O_15_ | Genipin-1-β-D-gentiobioside |  |
| 15 | 405.1385 | 5.1979 | C_17_H_25_O_11_ | Gardenoside |  |
| 16 | 415.2111 | 11.3140 | C_24_H_40_O_4_Na | HDCA |  |
| 17 | 427.1572 | 4.4463 | C_24_H_43_O_6_ | CA |  |

Table S2. Result from pathway analysis with MetPA. The column of Total expressed the total number of compounds in the pathway; the Hits expressed the actually matched number from the user uploaded data; the Raw p is the original p value calculated from the enrichment analysis; the Holm p is the p value adjusted by Holm-Bonferroni method; the FDR p is the p value adjusted using False Discovery Rate; the Impact is the pathway impact value calculated from pathway topology analysis.

| No. | Pathway name | Total | Expected | Hits | Raw p | Holm p | FDR | Impact |
| --- | --- | --- | --- | --- | --- | --- | --- | --- |
| 1 | Tryptophan metabolism | 41 | 0.43866 | 1 | 0.36076 | 1.0 | 1.0 | 0.15684 |
| 2 | Arginine and proline metabolism | 44 | 0.47076 | 5 | 5.751E-5 | 0.0046583 | 0.0046583 | 0.12973 |
| 3 | Alanine,aspartate and glutamate metabolism | 24 | 0.25678 | 1 | 0.22919 | 1.0 | 1.0 | 0.11392 |
| 4 | Glycine, serine and threonine metabolism | 32 | 0.34237 | 1 | 0.29397 | 1.0 | 1.0 | 0.03163 |
| 5 | Butanoate metabolism | 20 | 0.21398 | 1 | 0.19476 | 1.0 | 1.0 | 0.02899 |
| 6 | Pantothenate and CoA bosynthesis | 15 | 0.16049 | 1 | 0.1497 | 1.0 | 1.0 | 0.02041 |
| 7 | Histidine metabolism | 15 | 0.16049 | 1 | 0.1497 | 1.0 | 1.0 | 0.0 |
| 8 | Aminoacyl-tRNA biosynthess | 67 | 0.71683 | 2 | 0.15849 | 1.0 | 1.0 | 0.0 |
| 9 | Beta-Alanine metabolism | 19 | 0.20328 | 1 | 0.18593 | 1.0 | 1.0 | 0.0 |

Figure S2. Typical MS^1^to MS^3^ spectra of the metabolite with *m/z* 623.12573 obtained from rat urine.

Figure S3. PLS-DA loading plot based on urine metabolic profiling of the CG, PG and TG.
